# Trained ILCs confer adaptive immunity-independent protection against influenza

**DOI:** 10.1128/jvi.00532-25

**Published:** 2025-08-04

**Authors:** Wadzanai P. Mboko, Yuanyuan Wang, Weiping Cao, Ekramy E. Sayedahmed, Margarita Mishina, Amrita Kumar, Caitlin D. Bohannon, Sunni K. Patton, Sean D. Ray, Suresh D. Sharma, Rashmi Kumari, Justine S. Liepkalns, Adrian J. Reber, Ram P. Kamal, James McCoy, Samuel Amoah, Priya Ranjan, Mark Burroughs, Mili Sheth, Justin Lee, Dhwani Batra, Shivaprakash Gangappa, Ian A. York, Paul R. Knight, Jan Pohl, Suresh K. Mittal, Suryaprakash Sambhara

**Affiliations:** 1 Immunology and Pathogenesis Branch, Influenza Division, National Center for Immunization and Respiratory Diseases, Centers for Disease Control and Prevention, Atlanta, Georgia, USA; 2 Department of Comparative Pathobiology, Purdue University, West Lafayette, Indiana, USA; 3 Biotechnology Core Facility Branch, Division of Scientific Resources, National Center for Emerging and Zoonotic Infectious Diseases, Centers for Disease Control and Prevention, Atlanta, Georgia, USA; 4 Georgia State University, Atlanta, Georgia, USA; 5 Department of Anesthesiology, Jacobs School of Medicine and Biomedical Sciences, State University of New York at Buffalo, Buffalo, New York, USA; 6 Battelle Memorial Institute, Atlanta, Georgia, USA; 7 Department of Pathology and Laboratory Medicine, Emory University 189273, Atlanta, Georgia, USA; St. Jude Children's Research Hospital, Memphis, Tennessee, USA

**Keywords:** trained immunity, influenza, innate lymphoid cells, RNA-seq, adenoviral vectors

## Abstract

Seasonal influenza causes 290,000–650,000 deaths annually, with vaccination efficacy ranging from 10 to 60%. The emergence of drug-resistant and highly pathogenic avian influenza viruses underscores the urgent need for novel protective strategies. Epidemiological observations have long suggested that certain vaccines, such as Bacillus Calmette-Guérin (BCG), can provide protection against diverse pathogens (S. Biering-Sørensen, P. Aaby, N. Lund, et al., Clin Infect Dis 65:1183–1190, 2017, https://doi.org/10.1093/cid/cix525; M.-L. Garly, C. L. Martins, C. Balé, et al., Vaccine 21:2782–2790, 2003, https://doi.org/10.1016/s0264-410x(03)00181-6; C. A. G. Timmermann, S. Biering‐Sørensen, P. Aaby, et al., Trop Med Int Health 20:1733–1744, 2015, https://doi.org/10.1111/tmi.12614). While the cellular and molecular mechanisms underlying such protection remain incompletely understood, emerging research offers critical insights into innate immune system modulation (B. Cirovic, L. C. J. de Bree, L. Groh, et al., Cell Host Microbe 28:322–334, 2020, https://doi.org/10.1016/j.chom.2020.05.014; L. Kong, S. J. C. F. M. Moorlag, A. Lefkovith, et al., Cell Rep 37:110028, 2021, https://doi.org/10.1016/j.celrep.2021.110028; H. Mohammadi, N. Sharafkandi, M. Hemmatzadeh, et al., J Cell Physiol 233:4512–4529, 2018, https://doi.org/10.1002/jcp.26250; S. J. C. F. M. Moorlag, Y. A. Rodriguez-Rosales, J. Gillard, et al., Cell Rep 33:108387, 2021, https://doi.org/10.1016/j.celrep.2020.108387). We investigated whether a trained innate immune system with non-replicating adenoviruses could provide protection against diverse influenza virus strains. We demonstrated that replication-defective human adenoviruses can effectively train the innate immune system, conferring protective immunity in mice against multiple influenza virus strains, including H1N1, H3N2, H5N2, H7N9, and H9N2. In addition, bovine and chimpanzee adenoviruses can also activate human innate lymphoid cells (ILCs) and confer protection against challenge with influenza H3N2 virus in mice. Remarkably, this protection occurs in the complete absence of influenza-specific adaptive immune responses (influenza virus-specific hemagglutination-inhibiting antibodies, neutralizing antibodies, and influenza nucleoprotein-specific CD8 T cells). Key protective mechanisms include increased activation of ILC1, ILC2, and ILC3 populations, enhanced expression of interferon-stimulated genes (ISGs), upregulation of antiviral signaling pathways, and metabolic reprogramming of ILC subsets. Adoptive transfer experiments demonstrated that trained ILCs were sufficient to protect against influenza H1N1 infection in ILC-deficient mice. This research establishes a novel strategy for enhancing innate antiviral immunity, offering broad-spectrum protection against diverse influenza strains, a promising approach for not only pandemic preparedness but also against emerging infectious diseases. Training innate lymphoid cells through non-replicating adenoviral vectors represents a promising approach to enhancing broad-spectrum antiviral immunity, complementing traditional vaccination strategies.

**IMPORTANCE:**

The findings represent a potential game-changer for fighting influenza, which kills hundreds of thousands of people worldwide each year despite our best vaccination efforts. Current flu vaccines often provide limited protection because they must be reformulated annually to match circulating strains, and their effectiveness varies dramatically from year to year. The scientists discovered something remarkable: common adenoviruses (which typically cause mild cold-like symptoms) can essentially “train” our immune system’s first line of defense to recognize and fight off multiple types of flu viruses simultaneously. This protection works through a completely different mechanism than traditional vaccines—it does not rely on creating specific antibodies against flu proteins. Instead, the treatment activates special immune cells called innate lymphoid cells (ILCs), which act like the body’s rapid response team. These trained cells provide broad protection against various flu strains, including dangerous bird flu variants that could cause future pandemics. The significance lies in potentially creating a universal flu protection strategy that could work against unknown future flu strains, offering hope for better pandemic preparedness and reducing seasonal flu’s devastating global impact.

## INTRODUCTION

Seasonal influenza causes 290,000–650,000 deaths annually, with pandemic strains posing additional threats due to minimal pre-existing immunity (1). While vaccination remains the primary preventive measure, its efficacy varies substantially (10–60%) based on strain matching, egg-induced mutations in vaccine strains, repeated vaccination, and imprinting effects (2). The emergence of drug-resistant variants and highly pathogenic avian influenza viruses (H5N1 and H7N9) with mortality rates of 38–52% underscores the urgent need for novel protective strategies (3, 4).

Recent epidemiological studies have revealed that Bacillus Calmette-Guérin (BCG) vaccination confers broad protection against various infections, including severe acute respiratory syndrome coronavirus 2 (SARS-CoV-2), through “trained immunity” (TRIM) (5–9). This phenomenon operates independently of adaptive immunity and involves metabolic and epigenetic reprogramming of hematopoietic progenitors and myeloid cells (10–13). Similar immune enhancement has been observed with other microorganisms like *Cutibacterium acnes* (formerly known as *Corynebacterium parvum*) and non-replicating adenovirus particles, though their underlying mechanisms remain unclear (14–17).

Innate lymphoid cells (ILCs) are crucial regulators of mucosal immunity and have emerging roles in viral infections (18) (19–21). These cells are classified into three subsets based on their transcription factors and cytokine profiles. While recent studies have implicated ILCs in influenza-induced homeostasis and resolution, their potential in protective immunity against influenza remains largely unexplored (22).

Here, we investigated whether replication-defective adenoviral vectors—human Ad type 5 (HAd-ΔE1E3), chimpanzee Ad type 7 (ChAd-ΔE1E3), and bovine Ad type 3 (BAd-ΔE1E3)—could induce trained immunity against influenza virus infection. We examined the role of trained ILCs in mediating this protection and characterized their molecular signatures and metabolic changes through single-cell RNA sequencing (scRNA-seq) and pathway analyses.

## RESULTS

Initial studies from our group demonstrated that activating pathogen sensors like Retinoic Acid Inducible Gene-I (RIG-I) protects against diverse influenza viruses and Ebola virus, independent of adaptive immunity, likely through trained immunity induction (23–26). Given that adenoviral vectors activate innate immune responses (16) and activate human ILC *in vitro* (27), we investigated their potential to induce trained immunity against influenza viruses.

**Fig 1:**
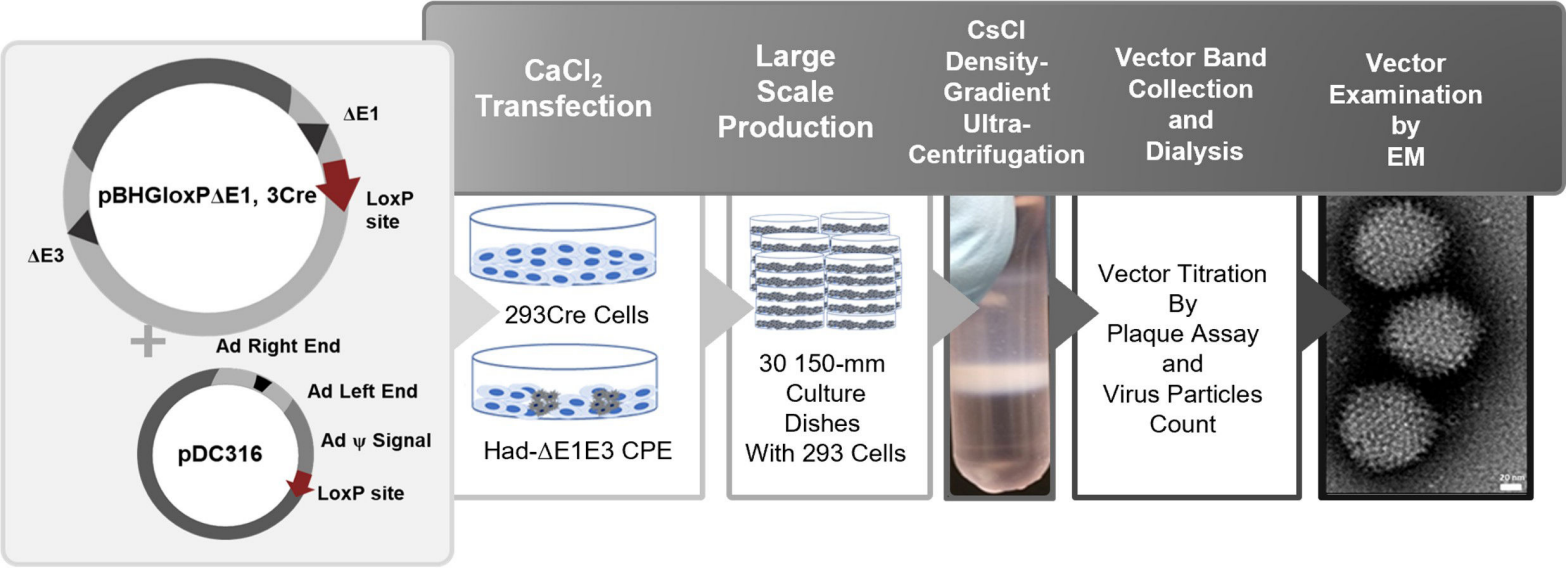
Generation, purification, titration, and visualization of replication-defective HAd-ΔE1E3 vectors. Replication-defective human adenovirus vectors lacking E1 and E3 regions (HAd-ΔE1E3) were generated by Cre recombinase-mediated recombination. Plasmids pBHGloxP-ΔE1, 3Cre, and pDC316 were transfected into 293Cre cells to obtain HAd-ΔE1E3 through Cre recombinase-mediated recombination. Vector was grown in thirty 150-mm culture dishes and purified using cesium chloride (CsCl) density-gradient ultracentrifugation and dialyzed to remove CsCl. Viral titers were determined by plaque assay on BHH2C cells, and virus particle concentrations were quantified by spectrophotometric analysis. E1- and E3-deleted chimpanzee Ad type 7 (ChAd-ΔE1E3) and bovine Ad type 3 (BAd-ΔE1E3) were grown in 293 and BHH3 cells, respectively. To assess vector morphology, vector particles (~90 nm) were visualized by transmission electron microscopy (TEM).

**Fig 2:**
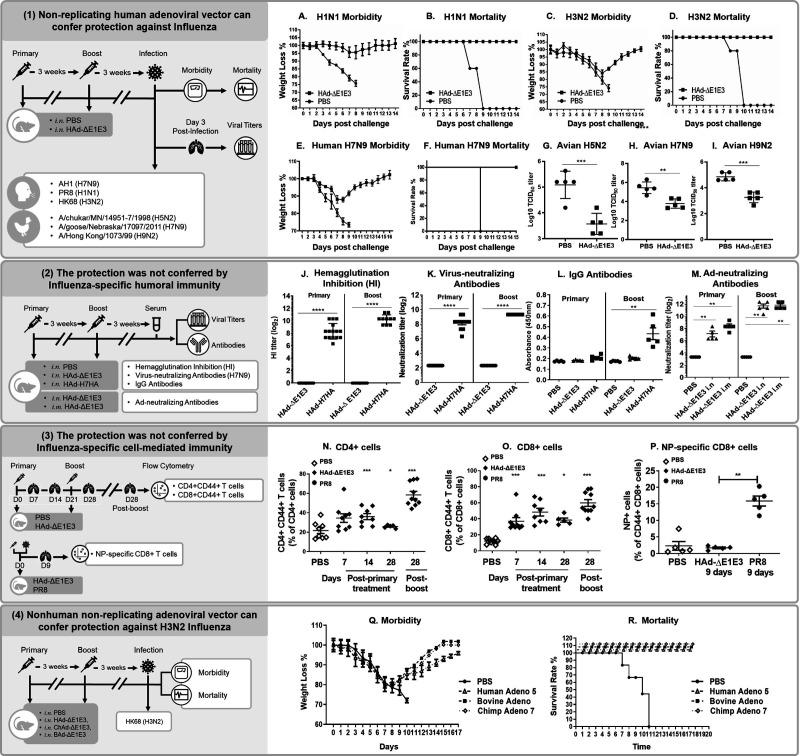
Non-replicating human adenoviral vector confers protective immunity to influenza virus in the absence of influenza-specific adaptive immunity.

We generated and characterized a non-replicating human adenoviral vector (HAd-ΔE1E3) (Fig. 1) and evaluated its protective efficacy in BALB/c mice using a prime-boost regimen. Following a challenge with human influenza viruses (H1N1, H3N2, or H7N9), HAd-ΔE1E3-treated mice showed 100% survival despite initial weight loss (Fig. 2A through F). Mice challenged with avian influenza strains (H5N2, H7N9, or H9N2) demonstrated significantly reduced lung viral titers compared to mock-inoculated controls, which either succumbed to infection or showed high viral loads (Fig. 2G through I).

To determine the mechanism of protection, we assessed both humoral and cellular immune responses. Unlike mice immunized with Ad expressing H7 hemagglutinin (HA), HAd-ΔE1E3-treated mice showed no hemagglutination inhibition activity (Fig. 2J), virus-neutralizing antibodies (Fig. 2K), or H7N9-specific IgG antibodies (Fig. 2L). Both groups developed comparable levels of Ad-neutralizing antibodies (Fig. 2M), indicating that protection was independent of influenza-specific antibodies.

Flow cytometric analysis of lung tissues revealed increased frequencies of activated CD8^+^ T cells by day 7, with both CD4^+^ and CD8^+^ T cell activation peaking at 14 days (36%) and 28 days post-boost (50–55%) (Fig. 2N and O). However, no influenza nucleoprotein-specific CD8^+^ T cells were detected in HAd-ΔE1E3-treated mice (Fig. 2P), a finding consistent across all influenza strain challenges. These results demonstrate that HAd-ΔE1E3-mediated protection operates independently of both influenza-specific humoral and cellular immunity.

We next investigated whether non-human adenoviral vectors could provide similar protection. Both bovine (BAd-ΔE1E3) and chimpanzee (ChAd-ΔE1E3) non-replicating vectors protected mice against A/Hong Kong/1/1968 (HK68), a H3N2 virus challenge, with all treated mice surviving despite weight loss, while PBS-treated controls succumbed to infection (Fig. 2Q and R). This demonstrates that trained immunity can be induced by diverse adenoviral vectors.

**Fig 3:**
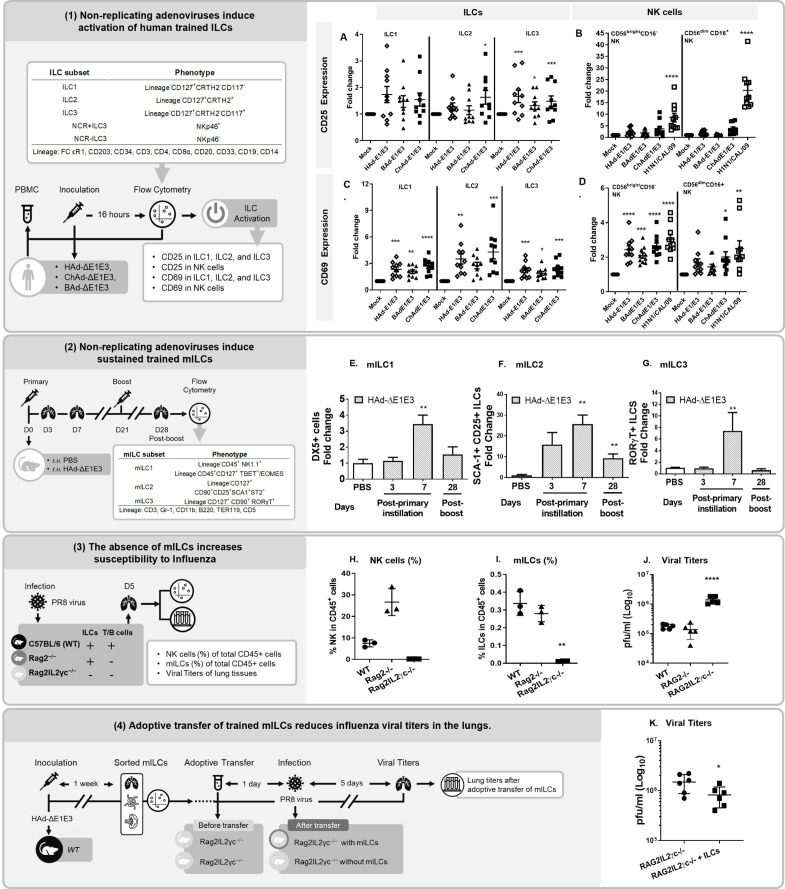
Non-replicating human adenoviral vectors train ILCs.

Given the established roles of ILCs in tissue homeostasis during influenza infection and protection against *Mycobacterium tuberculosis* (28), we investigated their potential involvement in adenovirus-induced trained immunity. Multi-parametric flow cytometry analysis of human peripheral blood mononuclear cells (PBMCs) exposed to HAdΔ-E1E3, ChAd-ΔE1E3, or BAd-ΔE1E3 (MOI = 10) revealed increased CD25 expression across ILC1, ILC2, and ILC3 populations (Fig. 3A), but not in NK cell subsets (Fig. 3B). All ILC subsets and CD56^bright^CD16 NK cells showed elevated CD69 expression in response to adenoviral treatment, while only ChAd-ΔE1E3 enhanced CD69 expression in CD56^dim^CD16^+^ NK cells (Fig. 3C and D).

*In vivo* analysis of trained ILC populations following intranasal HAd-ΔE1E3 administration revealed dynamic changes in lung ILC composition. DX5 + NK cells/ILC1s increased by day 7 (Fig. 3E), while ILC2s (CD90.2 + CD127+CD25+SCA-1+) showed dramatic expansion, reaching 15-fold and 25-fold increases over PBS controls at days 3 and 7, respectively (Fig. 3F). ILC3s (CD90.2^+^CD127^+^RORγT^+^) demonstrated a 6-fold increase by day 7 (Fig. 3G), indicating sustained expansion of trained ILCs, particularly ILC2s.

To establish the functional significance of trained ILCs, we compared PR8 virus infection outcomes in C57BL/6, Rag2^−/−^ (lacking T and B cells), and Rag2^−/−^IL2rγc^−/−^ (lacking T, B cells, and ILCs) mice. Rag2^−/−^IL2rγc^−/−^ mice showed marked reductions in NK and ILC populations across lungs, spleen, and bone marrow (Fig. 3H and I and Fig. S1) and significantly higher lung viral titers (~2×105 PFU/mL) compared to both C57BL/6 and Rag2^−/−^ mice (Fig. 3J). These findings demonstrate that the absence of trained ILCs increases susceptibility to influenza infection.

To directly demonstrate the protective role of trained ILCs, we adoptively transferred sorted ILCs (1 × 10^6^ cells/animal) from HAd-ΔE1E3-treated C57BL/6 mice into naive Rag2^−/−^ IL2γc^−/−^ mice. Following A/PR/8/34 (PR8), a H1N1 virus challenge, recipients of trained ILCs showed a statistically significant but modest reduction in lung viral titers compared to PBS-treated controls (Fig. 3K), indicating that trained ILCs contribute to protection against influenza infection.

**Fig 4:**
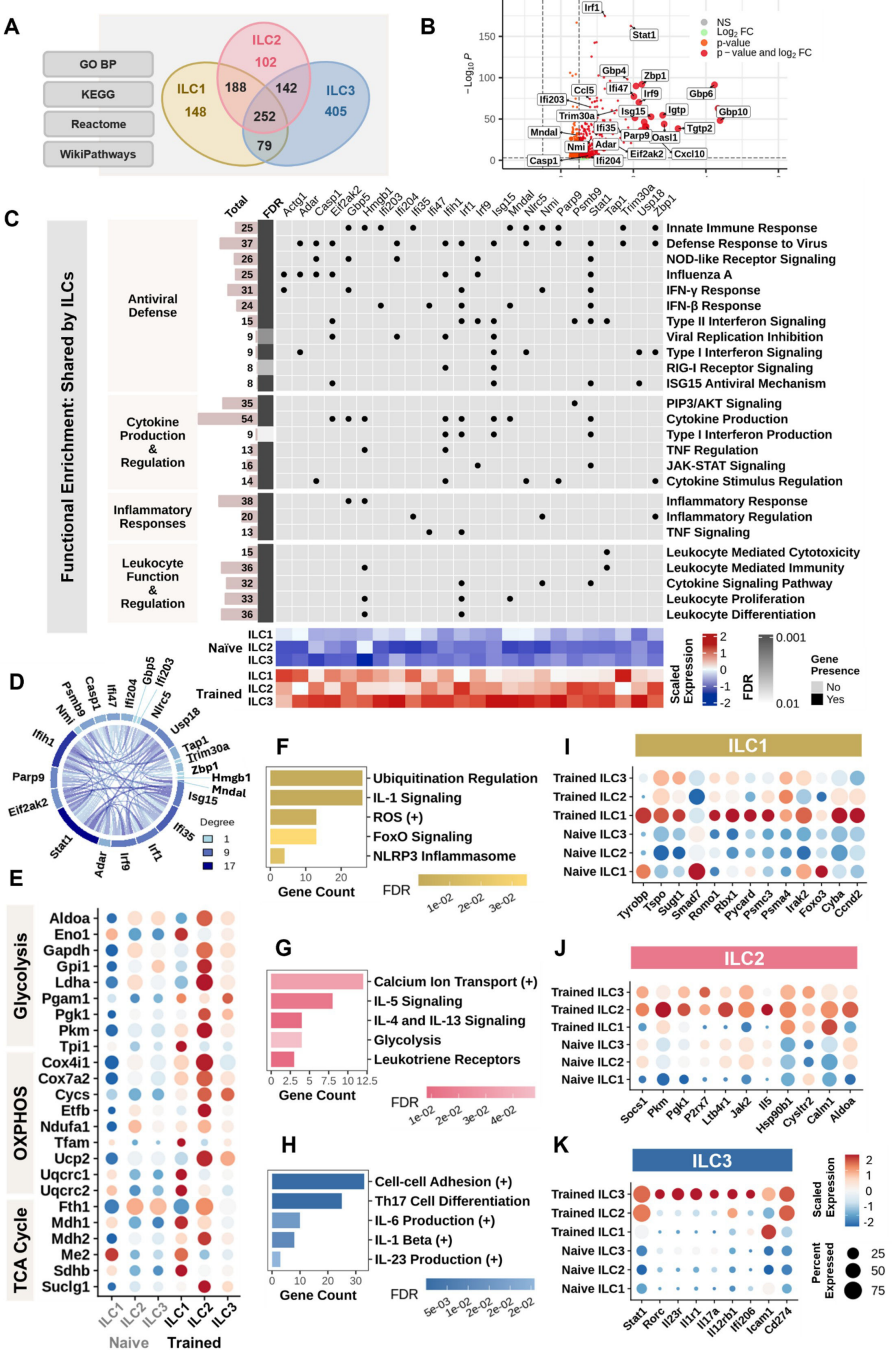
Transcriptome profile of trained mouse ILC in response to HAd-ΔE1E3 inoculation. (A) Venn diagram of shared and unique pathways enriched across ILC1, ILC2, and ILC3 based on GO Biological Processes, KEGG, Reactome, and WikiPathways. (B) Volcano plot of DEGs in trained ILCs, with log2 fold change (log2F) (x-axis) and -log10 *P*-value (y-axis). Genes are color-coded by significance, with red indicating high fold change and low *P*-value; genes with log2FC > 2 appear as enlarged dots. (C) Combined heatmap and bar plot of enriched pathways in ILCs. Rows represent pathways, columns represent genes, and dot color reflects false discovery rate (FDR) significance. Heatmap shows scaled gene expression across samples. (D) STRING network of 23 core genes in key pathways shown in C, with nodes as genes and edges as associations based on evidence. (E) Dot plot showing the expression of upregulated metabolic genes across ILC subsets. (F, G, and H) Bar plots of common pathways enriched in ILC1, ILC2, and ILC3, respectively, with gene count on the x-axis and bar color indicating FDR significance. (I, J, and K) Dot plots of gene expression across samples and ILC subtypes, with dot size showing the percentage of cells expressing each gene and color gradient indicating scaled expression levels. Dot plots E, I, J, and K share the same legend.

Since trained innate memory is supported by epigenetic modifications affecting gene expression analysis, we conducted scRNA-seq of sorted lung-infiltrating ILCs from HAd-ΔE1E3-treated mice and found both shared and distinct transcriptional signatures. Marker genes across sorted ILC subtypes are shown in Fig. S2A. We identified 915, 563, and 553 differentially expressed genes (DEGs in ILC1, ILC2, and ILC3 populations, respectively (adjusted *P* < 0.05, Tables S1 to S3 and Fig. S4), with 81 DEGs shared across all subsets (Fig. S2B). Functional enrichment analysis of GO BP (29), KEGG (30), Reactome (31), and WikiPathways (32) showed 665, 684, and 878 significantly enriched gene sets in ILC1, ILC2, and ILC3, respectively, with 252 pathways shared across all ILC subtypes and 409 pathways shared by two ILC subtypes (Fig. 4A).

Top DEGs, ranked by adjusted *P*-value and fold change (Fig. 4B), were multifunctional, predominantly involving ISGs and chemokines, such as CXCL9, CXCL10, and CCL5, suggesting critical to antiviral immunity (Fig. 4C). Innate immune response to viruses (GO:0051607, FDR = 1.84 × 10^−13^) was enriched through genes including ADAR, CASP1, IFIH1 (MDA5), ISG15, IRF1, IRF7, PARP9, STAT1, and ZBP1, suggesting enhanced viral recognition and response capabilities. This was further supported by enrichment of RIG-I receptor (KEGG:mmu04622, FDR = 3.5 × 10^−4^) and NOD-like receptor (KEGG:mmu04621, FDR = 7.15 × 10^−10^) signaling pathways.

Despite using non-replicating vectors, we observed activation of type I interferon signaling (GO:0060338, FDR = 3.3 × 10^−4^) and production (GO:0032481, FDR = 7.4 × 10^−4^) through IRF1, IRF7, IFNAR1, ISG15, IFIH1, and STAT2. The viral replication inhibition pathway (GO:0045071, FDR = 2.2 × 10^−4^) was enriched via IFI204, EIF2AK2, IFIH1, ISG15, ISG20, OAS3, BST2, and IFITM3, indicating establishment of an antiviral state. JAK-STAT signaling (KEGG:mmu04630, FDR = 7.88 × 10^−5^) and cytokine production (GO:0001819, FDR = 2.55 × 10^−15^) pathways were activated, alongside cytokine regulation (GO:0060760, FDR = 8.12 × 10^−7^) and TNF signaling (KEGG:mmu04668, FDR = 9.5 × 10^−5^). Additionally, IFN-γ response (GO:0034341, FDR = 2.25 × 10^−15^) and type II interferon signaling (WP1253, FDR = 1.97 × 10^−11^) were enriched through multiple genes including IFNγ, IFNγR1, IL12rb1, GBP5, GBP2, IRGM, IRF8, CCL5, and CXCl10. Additional details, including gene expression profile, pathway ID, genes, and statistical significance, are summarized in Fig. S3 and Table S4

STRING (33) protein-protein interaction analysis of 23 core genes from shared pathways revealed a highly connected network (confidence score 0.7) featuring 63 edges, an average node degree of 5.25, and a clustering coefficient of 0.72 (*P* < 1.0 × 10^−16^; Fig. 4D). Key interactions, such as ISG15-USP18, IRF1-STAT1, and ADAR-EIF2AK2, supported by multiple evidence channels (Fig. S5B), formed functional clusters with other genes induced in trained ILCs (Fig. S5A) with Stat1 and Isg15 emerging as central regulators of cytokine and interferon responses, highlighting coordinated interferon and cytokine signaling.

Alongside shared antiviral functions, each ILC subset exhibited distinct pathway enrichment profiles. ILC1 populations (Fig. 4F and G) showed enhanced ubiquitination regulation (GO:0031396, FDR = 7.90 × 10^−4^) through RBX1 and increased reactive oxygen species regulation (GO:2000379, FDR = 4.00 × 10^−3^) via CYBA, TSPO, and ROMO1. FOXO signaling (KEGG:mmu04068, FDR = 3.47 × 10^−2^) was modulated with changes in Foxo3 and Ccnd2, while IL-1 signaling (Reactome:MMU-9020702, FDR = 9.43 × 10^−11^) was enriched through IRAK2, PSMA4, and PSMC3, alongside NLRP3 inflammasome activation (Reactome:MMU-844456, FDR = 1.70 × 10^−2^) via Pycard and SUGT1.

ILC2s (Fig. 4H1) demonstrated enhanced calcium ion transport (GO:0051928, FDR = 3.25 × 10^−2^) through P2R × 7 and CALM1. This calcium signaling synergizes with IL-4/IL-13 (Reactome:MMU-6785807, FDR = 9.1 × 10^−3^) and IL-5 signaling (WikiPathway:WP151, FDR = 1.39 × 10^−2^) facilitated by upregulated HSP90B1, JAK2, SOCS1, and Il5. Elevated levels of glycolysis genes (PKM, PGK1, and ALDOA) and leukotriene receptor signaling genes (LTB4R1 and CYSLTR2; MMU-391906, FDR = 8.30 × 10^−3^) indicate enhanced metabolic activity and modulation of inflammatory responses.

ILC3 populations (Fig. 4J and K) showed enrichment in cell-cell adhesion (GO:0022409, FDR = 4.87 × 10^−9^) via ICAM1, CD274, and IL12rb1. Key inflammatory pathways were upregulated, including IL-1β (GO:0032731, FDR = 1.03 × 10^−2^) through ILR1 and STAT1 and IL-23 production pathways (GO:0032747, FDR = 2.58 × 10^−2^) through IL23R and IL12RB1. In addition, Th17 cell differentiation (KEGG:mmu04659, FDR = 4.59 × 10^−14^) was supported by the upregulation of RORC and IL17a, contributing to antiviral defense and enhancing the mucosal barrier.

Further transcriptional analysis of trained ILCs reveals distinct metabolic reprogramming in ILC subsets (Fig. 4E), with trained ILCs showing a strong shift toward glycolysis through the upregulation of ALDOA, ENO1, GAPDH, LDHA, PGK1, and PKM. Trained ILCs also show an increase in expression of target genes involved in oxidative phosphorylation (OXPHOS) and the TCA cycle, as shown in Fig. 4E. These data demonstrate that elevated glycolysis and mitochondrial bioenergetic pathways together fuel the metabolic demands and functional adaptations of trained ILCs (34).

While genes such as TYROBP, IRAK2, SOCS1, JAK2, and IL5 were identified as differentially upregulated in trained ILC subsets, we note that these genes also exhibit detectable expression in naïve ILCs. This pattern is consistent with the concept of trained immunity as a quantitative reprogramming process. The scRNA-seq data, generated from lungs at day 7 post-HAd-ΔE1E3 exposure, capture an early effector phase where transcriptional differences reflect enhanced activation rather than *de novo* gene expression. For instance, TYROBP and IRAK2 are basally expressed in ILC1s but further induced upon training, while SOCS1, JAK2, and IL5 show amplified expression in trained ILC2s. These findings support a model in which trained ILCs exist along a continuum of activation, exhibiting increased responsiveness rather than strict binary states.

In summary, these distinct molecular signatures reveal specialized functions: ILC1s primarily regulate cytokine signaling and antiviral defense, ILC2s exhibit metabolic reprogramming supporting tissue repair, and ILC3s coordinate inflammatory responses through IL-17/IL-23 signaling at mucosal surfaces.

## DISCUSSION

Our findings demonstrate that ILCs can be trained by adenoviral vectors to establish broad antiviral protection, revealing a novel mechanism of trained immunity distinct from BCG- or ChAdOx1 nCoV-19 vaccine-induced monocyte training (35, 36). While BCG vaccination has been shown to reduce SARS-CoV-2 seroprevalence and infection rates through monocyte-mediated trained immunity (37, 38), our work identifies ILCs as key mediators of vector-induced trained immunity.

Single-cell transcriptome analysis revealed that trained ILCs exhibit enhanced antiviral states across all subsets, characterized by upregulation of type I interferon-responsive genes. Notably, key upregulated factors include Adar1, which restricts viral replication through genomic editing (39); and MDA5 (encoded by IFIH1) (40), essential for sensing various viruses including SARS-CoV-2. The upregulation of Isg15, a key regulator of interferon signaling, underscores the antiviral state induced by vector training. Increased expression of transcription factors IRF1 and IRF7 (41, 42), both regulators of type I interferon responses, was observed, together with elevated levels of PARP9^51^ and ZBP1 (39), critical for innate antiviral defense. In addition, trained ILCs showed elevated levels of PARP enzymes and ZBP1, crucial for innate antiviral responses and inflammasome activation (43, 44).

Subset-specific analysis revealed distinct functional signatures: trained ILC1s showed enhanced IFNγ production, ILC2s increased IL-5, Amphiregulin, and GM-CSF expression, and ILC3s exhibited elevated IL-17a levels (45). Additionally, ILC3s displayed a broad spectrum of enriched pathways and elevated expression of ISG15, RTP4, NMI, PARP9, and DDX60, underscoring their antiviral roles at mucosal surfaces. These patterns align with their known immunomodulatory roles but now demonstrate enhanced functionality following training.

A metabolic switch towards glycolysis is reported to be a feature of immune proliferation and activation (34). This elevated transcriptional upregulation of glycolysis enzymes in the trained ILCs might be necessary to prepare the cells to respond to both related and unrelated pathogens rapidly.

While characterization of specific ILC subsets mediating protection presents technical challenges due to their low frequency and the complexity of isolation procedures, our findings suggest that training ILCs via non-replicating adenoviral vectors may represent a practical approach for enhancing broad-spectrum antiviral immunity. This approach could be particularly valuable in emerging epidemic or pandemic situations where pathogen-specific interventions are unavailable. To circumvent potential interference from pre-existing immunity to human adenoviruses, non-human adenoviral vectors could be employed as training agents.

While we focused on lung viral titers as the primary measure of protective efficacy, upper respiratory tract viral shedding was not assessed. Future studies incorporating nasal wash or throat swab sampling will be important for evaluating the impact of trained ILCs on viral transmission. Additionally, although BALB/c and C57BL/6 mice differ in their baseline ILC composition and cytokine responsiveness, our findings suggest that adenoviral vector-induced ILC training occurs across both genetic backgrounds. However, no direct, head-to-head comparison of trained ILC responses has been performed at single-cell resolution, and future work addressing this gap could clarify how host genetics influence the magnitude and character of innate immune memory.

This work establishes ILC training as a novel strategy for enhancing innate antiviral immunity, complementing our understanding of trained immunity beyond the well-characterized BCG-monocyte axis. Future studies should focus on optimizing vector design and delivery methods and exploring the utility of the ligands of pathogen sensors and small molecules to induce and maximize training efficacy while ensuring safety, potentially paving the way for innate immune system-based interventions.

## MATERIALS AND METHODS

### Generation and characterization of adenovirus vectors

We used replication-deficient HAd-ΔE1E3 (46) and ChAd-ΔE1E3 (from S. Worgall, Weill Cornell Medicine) grown in 293 cells, and BAd-ΔE1E3 grown in BHH3 cells. HAd-H7HA was constructed by inserting the full-length HA gene from A/Anhui/1/2013 (AH1) H7N9 virus into HAd-ΔE1E3. Vector genomes were confirmed by restriction enzyme digestion and sequencing. H7HA expression was verified by immunoblot analysis of infected HEK 293 cells using ferret anti-A/Netherland/219/03 (H7N9) antibody (47). All vectors were purified by cesium chloride density-gradient ultracentrifugation and titrated by plaque assay.

### Influenza viruses

We used A/Puerto Rico/8/34 (PR8, H1N1), A/Anhui/1/2013 (AH1, H7N9), mouse-adapted A/Hong Kong/1/68 (HK68, H3N2), A/Taiwan/1/86 (TW86, H1N1), A/California/08/2009, A/chukar/MN/14951-7/1998 (H5N2), A/goose/Nebraska/17097/2011 (H7N9), A/Hong Kong/1073/1999 (H9N2), and SH2/PR8 (H7N9). Viruses were propagated in embryonated chicken eggs, harvested after 48 h, and stored at −80 °C.

### Flow cytometry

Human PBMCs were isolated using CPT tubes (BD Bioscience) with IRB approval. Cells were incubated with adenovirus vectors (1:1000) for 16 h and analyzed using fluorochrome-conjugated antibodies against CD56, CD3, CD16, CD127, CD117, CRTH2, CD69, CD25, and lineage markers. Mouse lung tissues were processed using the Lung Dissociation Kit (Miltenyi Biotec). ILCs were identified using antibodies against lineage markers, SCA-1, CD45, CD90.2, RORγt, CD127, CD25, and DX5. Analysis was performed on a BD LSRFortessa.

### Animal studies

Five- to six-week-old, female BALB/c mice (Jackson Laboratories, Bar Harbor, ME, *n* = 5/group) received intranasal inoculations of PBS or 2.2 × 10^10^HAd-ΔE1E3 twice at 3-week intervals. Three weeks post-boost, mice were challenged with influenza viruses: 100 MID_50_ of avian H5N2, human and avian H7N9, or avian H9N2; 5 LD_50_ of PR8; or 2 LD_50_ of HK68. For adoptive transfer of ILC and initial characterization of the mice (Fig. S1), C57BL/6, Rag2^−/−^, and Rag2^−/−^ IL2γc^−/−^ mice were infected with PR8 (25 MID_50_). ILCs from HAd-ΔE1E3-treated C57BL/6 mice were transferred to Rag2^−/−^IL2γc^−/−^ mice (1 × 10^6^ cells/mouse) before PR8 challenge.

### Virus titration

Three days post-challenge with avian H5N2, avian H7N9, and avian H9N2 influenza viruses, the lungs were collected, and homogenates were made and stored at −80° C to determine lung viral titers. Lung homogenates were thawed, serially diluted, and plaque assays performed on MDCK cells using agarose overlay medium containing TPCK-trypsin. Plaques were quantified after crystal violet staining at 72 h.

### scRNA-seq and analysis

ILC1, ILC2, and ILC3 populations were isolated from the lungs of HAd-ΔE1E3-treated (7 days post-inoculation) and naïve mice. ScRNA-seq was performed using Chromium Single Cell 3ʹ v3 kits (10x Genomics) and sequenced on Illumina HiSeq with >27,000 read pairs per cell. Data were processed using Cell Ranger v6.0 and Seurat v5 in R. (48) Cells were filtered based on UMI count (>500), gene count (>250), gene-to-UMI log10 ratio (>0.80), and mitochondrial content (<20%). Doublets were removed using DoubletFinder (threshold 0.8) (49,50). Median captured cells were 815 per sample, with 2,169 genes expressed per cell. Differential expression analysis used the Wilcoxon rank sum test (adjusted *P* < 0.05, |log fold change| > 0.25) provided in Seurat v5. Functional enrichment analysis was performed using STRING v12.0 (33) with high-confidence associations (0.7). STRING networks were analyzed for Gene Ontology (29), KEGG (30), Reactome (31), and WikiPathways (32), reporting gene count and FDR. Data were analyzed in R and documented at https://github.com/yyw-informatics/ILC_trained_immunity.git.

### Serological assays and analysis

HI assays were performed using horse RBC with RDE-treated serum and four hemagglutination units of SH2/PR8 virus. For microneutralization, RDE-treated serum was incubated with SH2/PR8 (2 × 10^3^ TCID_50_/mL) before adding MDCK cells. Detection used biotinylated anti-nucleoprotein antibody and streptavidin-HRP. Ad neutralization was assessed by incubating heat-inactivated serum with human Ad (100 PFU) and BHH-2C cells. Enzyme-linked immunosorbent assay plates were coated with H7HA (1 µg/mL) or SH2/PR8 H7N9 virus (50 HAU), and serum IgG was detected using HRP-conjugated secondary antibody (47). Data are mean ±SEM.

### Statistical analysis

The data were analyzed using GraphPad Prism 5.0 using a non-parametric Mann-Whitney U test. Statistical significances are represented by * (*P* ≤ 0.05), ** (*P* ≤ 0.01), *** (*P* ≤ 0.001), and **** (*P* ≤ 0.0001).

## Data Availability

The data sets generated and analyzed during the current study are available from the corresponding author on reasonable request. Processed data files, including gene expression matrices and statistical analysis outputs, are available in the supplemental material. Reagents and materials used in this study are available from the corresponding author under a material transfer agreement with the institution. Mouse strains and cell lines used are commercially available or can be obtained from the indicated repositories as described in Materials and Methods.

## SUPPLEMENTAL MATERIAL

Figure S1 jvi.00532-25-s0001.tif Induction of NK and ILC in C57BL/6, Rag2^−/−^, and Rag2IL2γc^−/−^ mice after PR8 infection.

Figure S2 jvi.00532-25-s0002.tif Marker genes for ILC subtypes and differentially expressed gene intersections among ILC subtypes.

Figure S3 jvi.00532-25-s0003.tif Gene expression of antiviral genes, ISGs, and chemokines induced in ILCs trained with HAd-ΔE1E3.

Figure S4 jvi.00532-25-s0004.tif Volcano plots of DEGs found in ILC1, ILC2, and ILC3.

Figure S5 jvi.00532-25-s0005.tif STRING network of genes induced in ILCs trained with HAd-ΔE1E3.

Supplemental legends. jvi.00532-25-s0006.docx Legends for Fig. S1 to S5 and Tables S1 to S4.

Table S1 jvi.00532-25-s0007.xlsx DEGs in ILC1.

Table S2 jvi.00532-25-s0008.xlsx DEGs in ILC2.

Table S3 jvi.00532-25-s0009.xlsx DEGs in ILC3.

Table S4 jvi.00532-25-s0010.xlsx Enriched pathways shared among ILCs.

ASM does not own the copyrights to Supplemental Material that may be linked to, or accessed through, an article. The authors have granted ASM a non-exclusive, world-wide license to publish the Supplemental Material files. Please contact the corresponding author directly for reuse.
