# Supplementary figures and images for: Trained ILCs confer adaptive immunity-independent protection against influenza
Source: J Virol. 2025 Aug 4;99(9):e00532-25. doi: 10.1128/jvi.00532-25 (PMC12455922; doi:10.1128/jvi.00532-25)

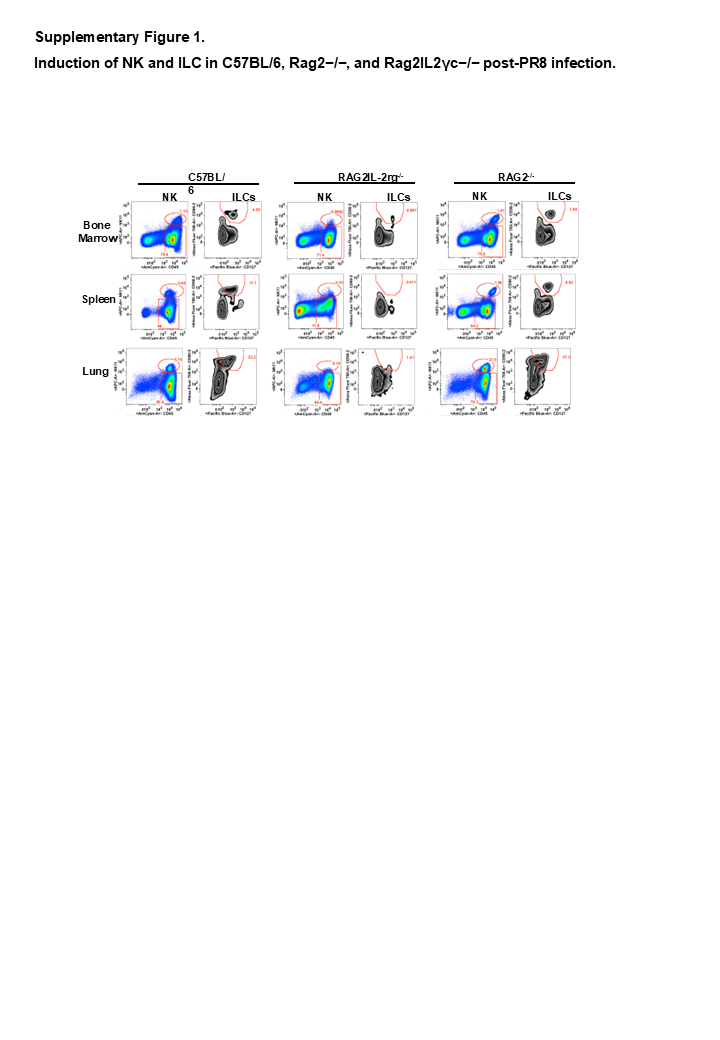

Supplement: Figure S1 — Induction of NK and ILC in C57BL/6, Rag2−/−, and Rag2IL2γc−/− mice after PR8 infection. [file jvi.00532-25-s0001.tif]

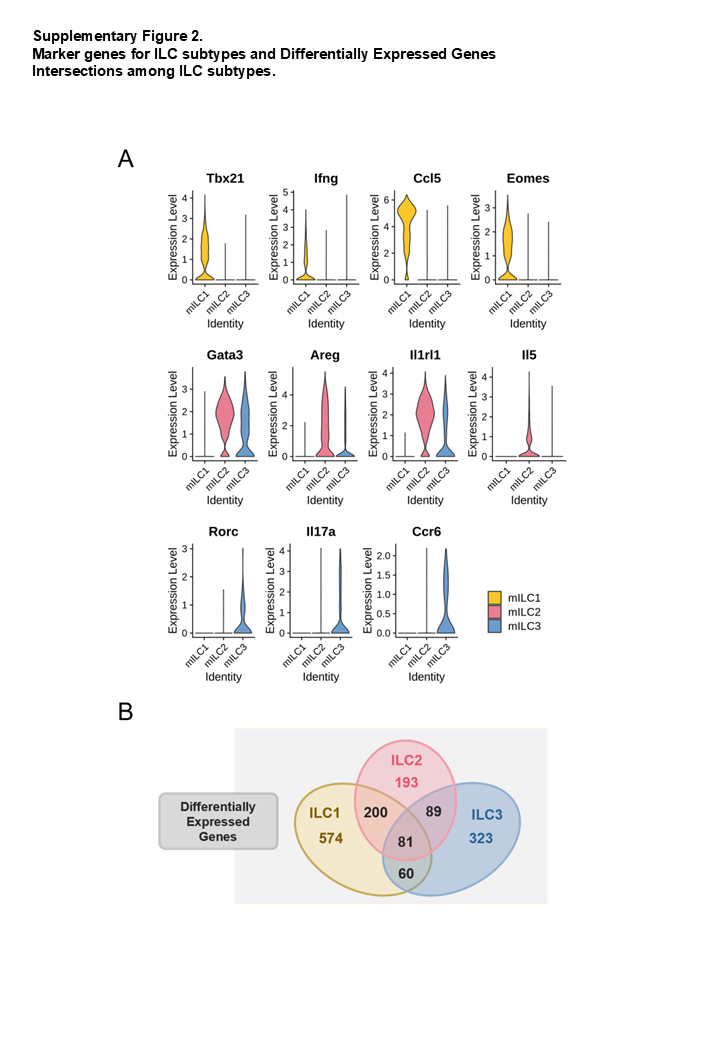

Supplement: Figure S2 — Marker genes for ILC subtypes and differentially expressed gene intersections among ILC subtypes. [file jvi.00532-25-s0002.tif]

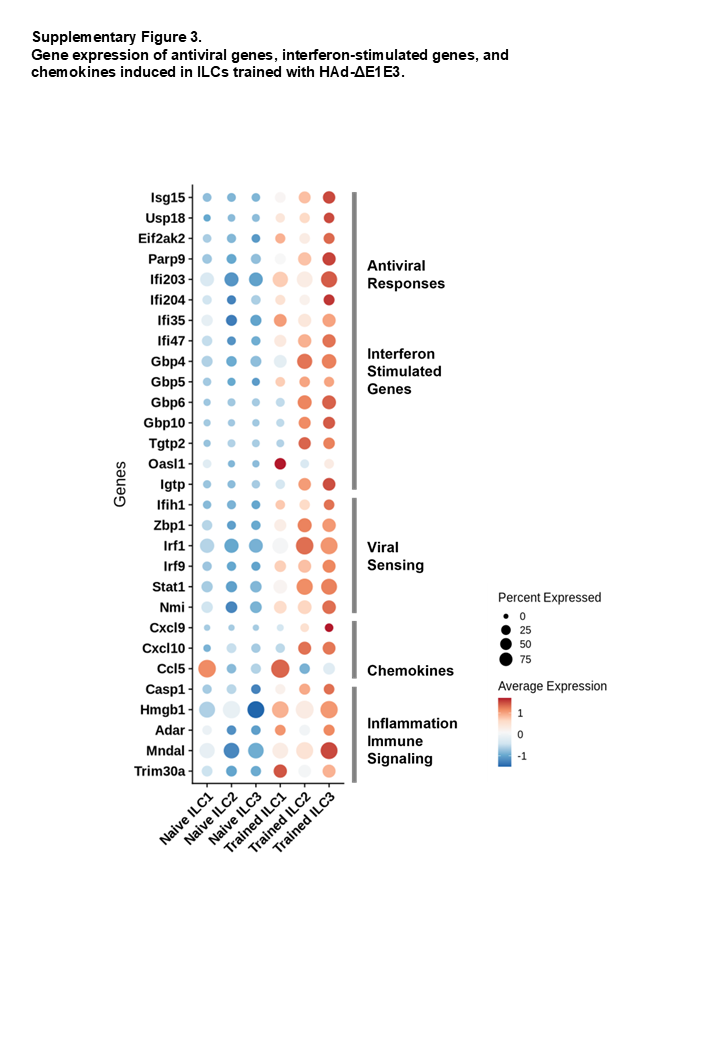

Supplement: Figure S3 — Gene expression of antiviral genes, ISGs, and chemokines induced in ILCs trained with HAd-ΔE1E3. [file jvi.00532-25-s0003.tif]

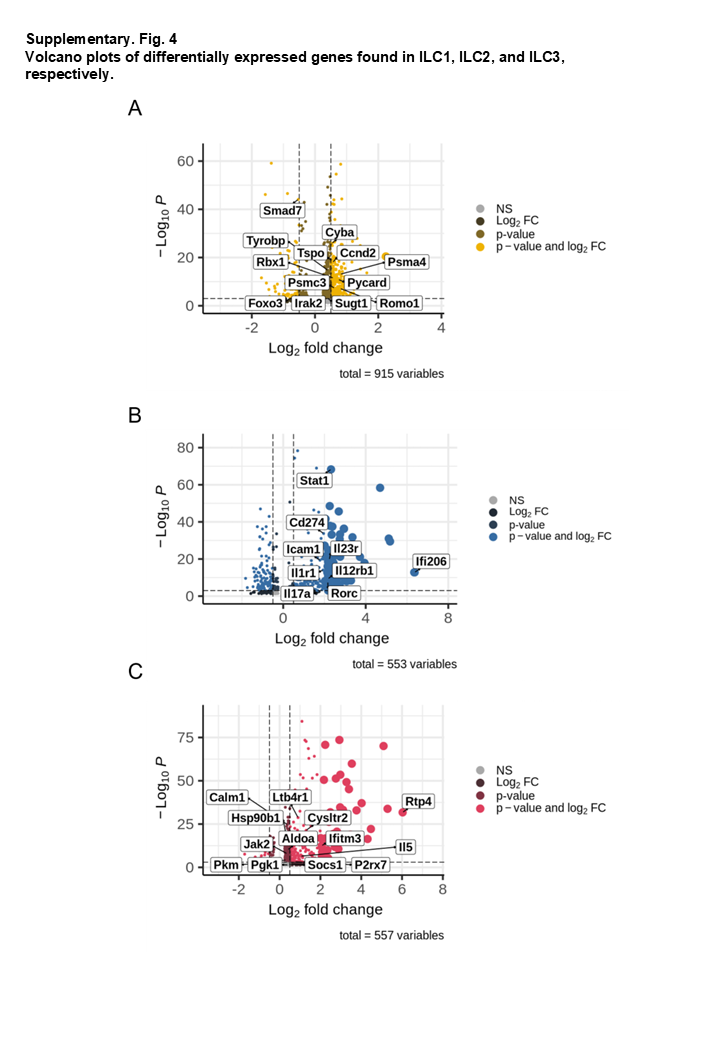

Supplement: Figure S4 — Volcano plots of DEGs found in ILC1, ILC2, and ILC3. [file jvi.00532-25-s0004.tif]

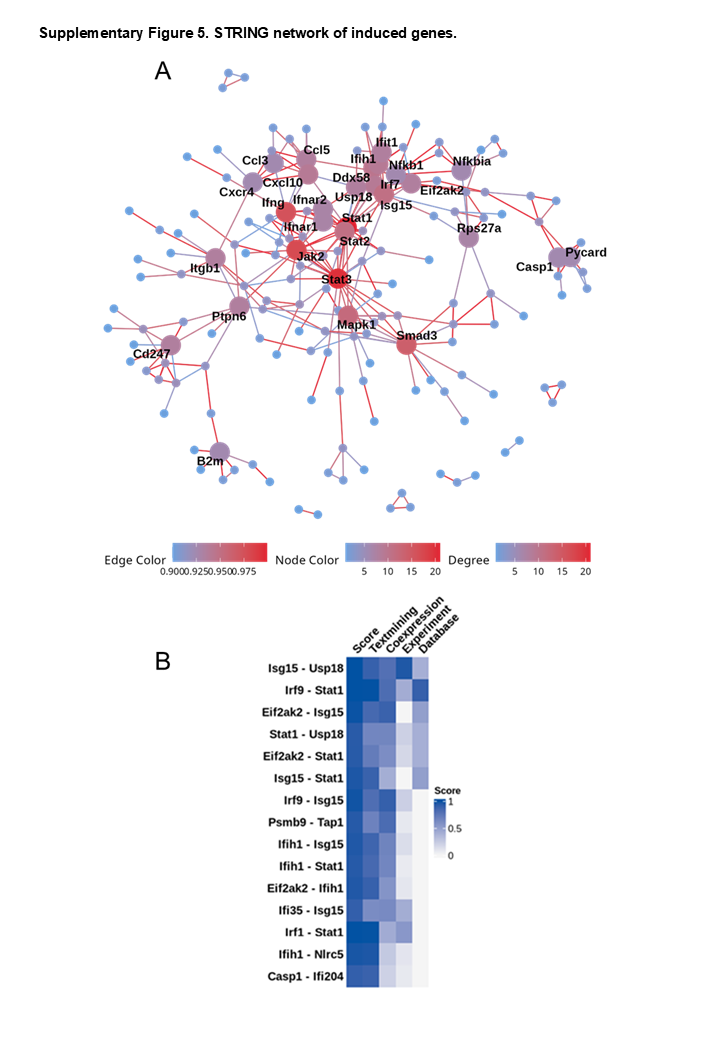

Supplement: Figure S5 — STRING network of genes induced in ILCs trained with HAd-ΔE1E3. [file jvi.00532-25-s0005.tif]
