# Supplementary material for: Trained ILCs confer adaptive immunity-independent protection against influenza
Source: J Virol. 2025 Aug 4;99(9):e00532-25. doi: 10.1128/jvi.00532-25 (PMC12455922; doi:10.1128/jvi.00532-25)
Supplement: Supplemental legends. — Legends for Fig. S1 to S5 and Tables S1 to S4. [file jvi.00532-25-s0006.docx]

**Supplementary Figure and Table Legends**

**Supplementary Fig. 1. Induction of NK and ILC in C57BL/6, Rag2−/−, and Rag2IL2γc−/− post-PR8 infection. Five to six-week-old female** C57BL/6, Rag2−/−, and Rag2IL2γc−/− mice were infected with PR8 virus (25 MID_50_, i.n.). Bone marrow, spleen, and lung tissues were harvested 5 days post-infection. Single-cell suspensions were prepared and stained with antibodies to identify NK and total ILC. The percentage of Lin-NK1.1+ NK cells in CD45+ cells and percentage of total CD127+CD90.2+ non-NK ILCs in Lin-NK1.1-CD45+ cells are displayed.

**Supplementary Fig. 2. Marker genes for ILC subtypes and Differentially Expressed Genes Intersections among ILC subtypes. A.** Violin plots showing the expression distribution of marker genes for mILCs. **B.** Venn diagram of differentially expressed genes (DEGs) in ILC populations.

**Supplementary Fig. 3. Gene expression of antiviral genes, interferon-stimulated genes, and chemokines induced in ILCs trained with HAd-ΔE1E3.** Dot plots of gene expression across samples and ILC subtypes, with dot size showing the percentage of cells expressing each gene and color gradient indicating scaled expression levels.

**Supplementary Fig. 4 Volcano plots of differentially expressed genes found in ILC1, ILC2, and ILC3, respectively.** The x-axis represents the log2 fold change of gene expression between the conditions, with positive values indicating upregulation and negative values indicating downregulation. The y-axis shows the -log10 p-value, where higher values indicate greater statistical significance.

**Supplementary Fig. 5. STRING network of genes induced in ILCs trained with HAd-ΔE1E3.**

**A.** Network was constructed using induced genes in trained ILCs. The high confidence network (confidence = 0.7) consists of 278 nodes and 583 edges, representing key interactions among genes identified through STRING analysis. Nodes with degree >5 are enlarged and labeled. Average node degree: 4.19; average local clustering coefficient: 0.447. PPI enrichment p-value: <1.0e-16. **B.** Heatmap of STRING evidence scores for top gene interactions, with darker colors indicating stronger support from sources of evidence.

**Supplementary Tables. Differentially Expressed Genes per ILC Subtype and Shared Enriched Pathways among ILC Populations**

Table 1. Differentially expressed genes in ILC1

Table 2. Differentially expressed genes in ILC2

Table 3. Differentially expressed genes in ILC3

Table 4. Enriched Pathways shared among ILCs.
